# Severe Corrosive Gastritis Caused by Accidental Ingestion of Mildly Alkaline Calcium Chloride Desiccant: A Case Report

**DOI:** 10.1002/deo2.70185

**Published:** 2025-11-14

**Authors:** Ayaka Mitomo, Soojin Kim, Ryota Omae, Ruka Kinjo, Ryoma Morimoto, Naotaka Nakama, Yuki Nagata

**Affiliations:** ^1^ Gastroenterology, Chubu Tokushukai Hospital Okinawa Japan; ^2^ Kidney Disease and Transplant Center Shonan Kamakura General Hospital Kamakura Kanagawa Japan

**Keywords:** alkaline, calcium chloride, corrosive gastritis, ulcer, upper gastrointestinal endoscopy

## Abstract

A woman in her 90s with dementia accidentally ingested an unknown amount of calcium chloride–containing desiccant (pH 7–8). Computed tomography (CT) revealed circumferential gastric wall thickening, and upper gastrointestinal endoscopy revealed ulcers with necrotic material extending from the cardia to the greater curvature of the pyloric region, along with erosion of the posterior wall of the descending duodenum. The patient was diagnosed with severe corrosive gastritis and was treated with omeprazole, sucralfate, and antibiotics (SBT/ABPC). Upper gastrointestinal endoscopy performed on hospitalization days 15, 29, and 80 revealed gradual improvement of the ulcers without stenosis, not requiring treatment. Severe injuries caused by the ingestion of strongly alkaline agents, such as chlorine‐based bleaches, are well known. However, as in this case, even mildly alkaline agents can cause severe injuries depending on the amount ingested, presence of solid components, and time elapsed after ingestion. Therefore, it is crucial to consider this possibility in clinical practice.

## 1 Introduction

Corrosive gastritis is caused by the ingestion of corrosive substances and presents with a wide range of clinical manifestations depending on the pH, concentration, amount, and duration of exposure. Alkaline substances tend to cause severe injuries due to saponification and liquefactive necrosis, which facilitate deep tissue penetration, including into the muscularis propria [1]. Herein, we report a case of severe corrosive gastritis caused by the accidental ingestion of calcium chloride, a mildly alkaline substance widely used as a desiccant in foods, pharmaceuticals, and dehumidifiers.

## 2 Case Report

**FIGURE 1:**
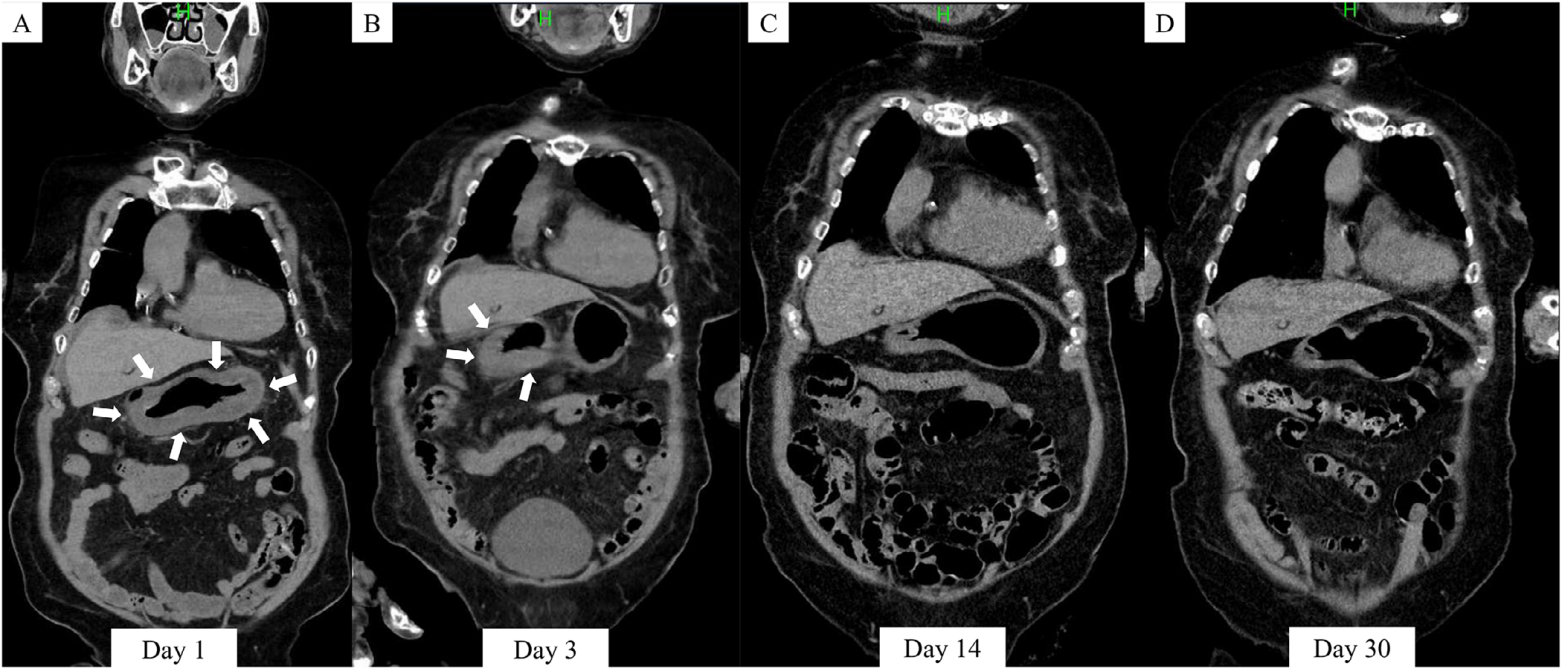
Computed tomography (CT) findings. (A) Abdominal CT revealed circumferential edematous thickening of the gastric wall. No evidence of pneumomediastinum or free air in the abdominal cavity was observed. (B–D) Follow‐up CT demonstrated gradual improvement in the circumferential gastric wall thickening.

The patient was a woman in her 90s with a history of dementia, multiple cerebral infarctions, and hypertension. Two days before admission, the patient experienced vomiting and difficulty moving, which resulted in an inability to eat. The patient was examined at a local clinic, where blood tests revealed a corrected calcium level of 12.1 mg/dL. A calcium chloride–containing desiccant was found spilled near the patient, raising suspicion of accidental ingestion, leading to hypercalcemia and gastrointestinal mucosal injury. The patient was subsequently referred to the emergency department. The initial vital signs were as follows: blood pressure, 129/91 mmHg; heart rate, 100 beats/min; respiratory rate, 20 breaths/min; body temperature, 37.1°C; and oxygen saturation, 95% on room air. Physical examination revealed lethargy; however, her abdomen was flat, soft, and non‐tender. The patient denied oral pain, and no mucosal burns or ulcers were observed. There were no signs of abdominal distension, severe diarrhea, or peritoneal irritation suggestive of distal gastrointestinal injury. Venous blood gas analysis showed a pH of 7.43, CO_2_ of 37.1 mmHg, HCO_3_ of 24.4 mmHg, and a lactate level of 1.45 mmol/L. Laboratory results included leukocytes at 20,400/µL, lactate dehydrogenase (LDH) at 234 U/L, blood urea nitrogen at 28.4 mg/dL, creatinine at 0.76 mg/dL, potassium at 3.4 mmol/L, corrected calcium at 10.3 mg/dL, C‐reactive protein (CRP) at 13.13 mg/dL, and d‐dimer at 4.9 µg/mL. Abdominal CT showed diffuse gastric wall edema without pneumomediastinum, free air, or signs of small bowel necrosis (Figure 1A).

**FIGURE 2:**
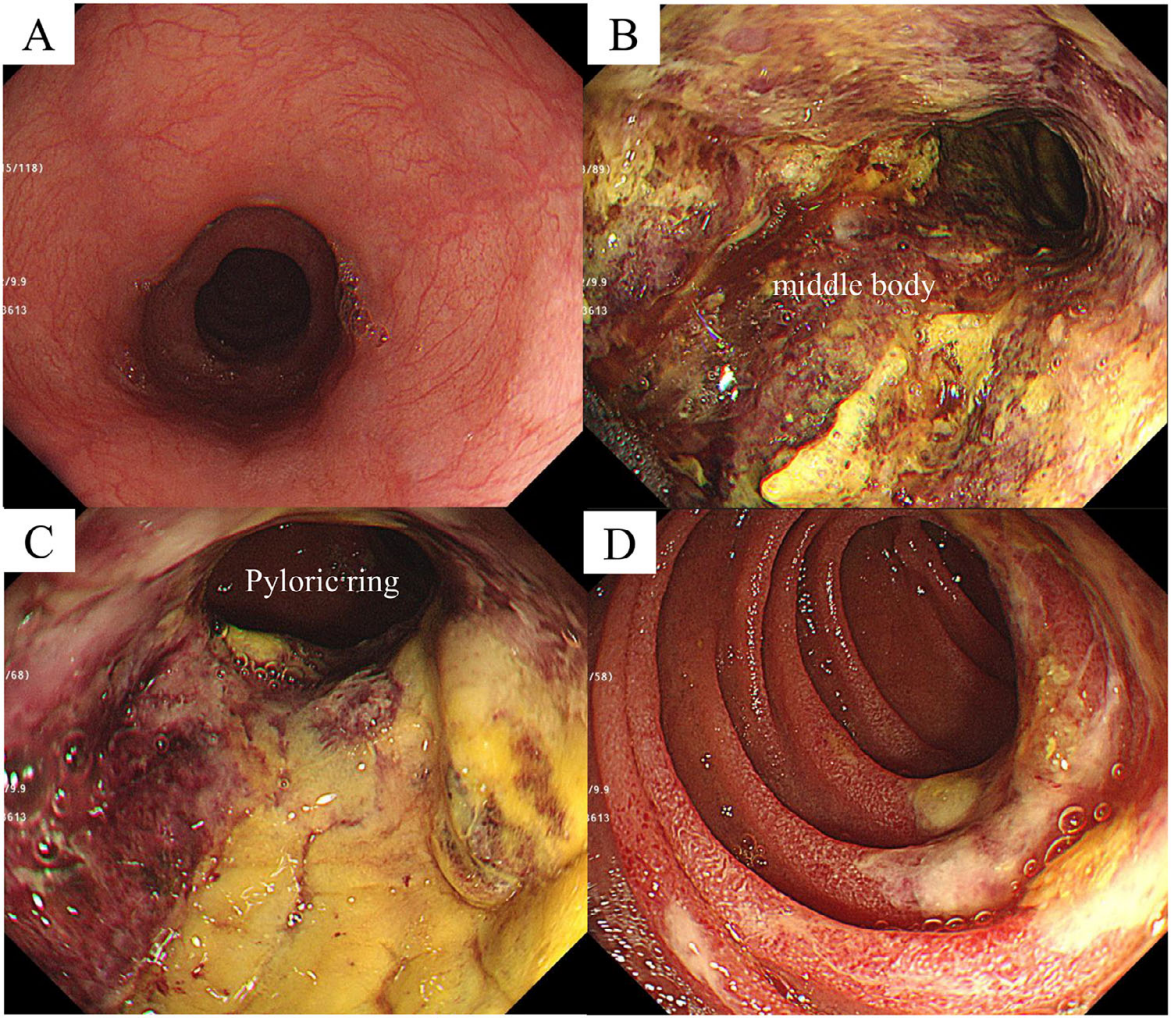
Upper gastrointestinal endoscopy findings at admission. (A) No significant abnormalities were observed in the thoracic esophagus. (B and C) Circumferential ulcers with necrotic material were observed from the cardia to the greater curvature of the pyloric region. (D) Erosions were observed on the posterior wall of the descending part of the duodenum.

Emergency upper gastrointestinal endoscopy revealed no abnormalities such as erosions in the esophagus, as shown in Figure 2A. As demonstrated in Figure 2B,C, circumferential ulcers with necrotic debris were observed extending from the cardia to the greater curvature of the pyloric region. In the duodenum, as shown in Figure 2D, mild erosions were noted on the posterior wall of the descending portion, which were less severe than those in the stomach (Figure 2). The esophagus was unaffected. On the basis of CT findings of gastric wall thickening, differential diagnoses included ischemic gastritis, phlegmonous gastritis, acute gastric mucosal lesions (AGML), and gastric anisakiasis. Ischemic gastritis often presents with longitudinal or irregular ulcers, mucosal edema, erythema, and bleeding. However, the absence of hypotension, elevated d‐dimer, and ischemic endoscopic changes made this unlikely. Phlegmonous gastritis typically involves mucosal edema with purulent exudates, which were not seen. AGML is marked by irregular ulcers of varying depth and size, which were absent. No Anisakis larvae were detected. These findings supported a diagnosis of severe corrosive gastritis due to ingestion of a mildly alkaline substance.

The presence of extensive necrosis indicated a high risk of perforation because the injury was caused by an alkaline substance, which is known to cause progressive tissue liquefaction. The patient was admitted to a high‐care unit for close monitoring and conservative management, including fasting, intravenous fluids, omeprazole, sucralfate, and antibiotics (SBT/ABPC) administration. Close monitoring included frequent assessments for the onset of abdominal pain, elevations in LDH and lactate levels, and imaging to detect changes, such as increased ascites or mediastinal abnormalities.

**FIGURE 3:**
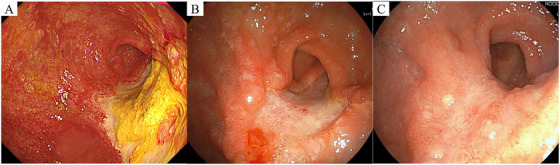
Follow‐up upper gastrointestinal endoscopy findings. (A) Day 15: Deep circumferential ulcers with white coating from the antrum to the angular incisure, showing improvement. No stenosis; smooth scope passage. (B) Day 29: Ulcers persisted, but necrotic material resolved. No stenosis. (C) Day 80: The ulcers showed improvement with evidence of scarring. Mild pyloric stenosis; however, the scope passed easily.

Follow‐up CT on hospitalization day 3 showed improvement in circumferential gastric wall thickening (Figure 1B). A peak in CRP levels was confirmed on Day 6, and in the absence of abdominal pain or signs of peritonitis, a liquid diet was initiated. Subsequently, upper gastrointestinal endoscopy on Day 15 revealed no evidence of perforation or significant structure and demonstrated improvement in the gastric ulcers. Therefore, the patient's diet was advanced to a semi‐solid rice porridge on the same day. As no abdominal symptoms developed thereafter, a full porridge diet was introduced on hospitalization day 21. A second upper gastrointestinal endoscopy performed on Day 15 revealed circumferential ulcers with thick white coatings extending from the antrum to the posterior wall of the angular incisure. Although the ulcers were deep, improvement was observed, and no stenosis was noted, allowing smooth passage of the scope (Figure 3A). A third endoscopy on Day 29 revealed the disappearance of necrotic material, with residual ulcers showing improvement and no stenosis (Figure 3B). The patient was discharged to a care facility on Day 49 of hospitalization. Outpatient follow‐up endoscopy performed on Day 80 after ingestion showed scarring of the gastric ulcers, with mild stenosis at the pylorus. However, the scope still passed easily (Figure 3C).

## 3 Discussion

Severe corrosive gastritis due to alkaline substances is typically caused by strongly basic agents such as sodium hydroxide (caustic soda), potassium hydroxide, and sodium hypochlorite, commonly present in drain cleaners and bleach. These substances possess a pH of 11–14, classifying them as strongly alkaline and capable of inducing deep mucosal injury. In contrast, the ingested agent in this case, calcium chloride, is mildly alkaline and widely used as a desiccant in food, pharmaceuticals, and dehumidifying products. Manufacturer inquiries confirmed the desiccant's pH to be approximately 7–8. Although calcium chloride has a distinctly bitter taste that generally limits large intake, accidental ingestion of toxic amounts may occur, especially in individuals with dementia.

**FIGURE 4:**
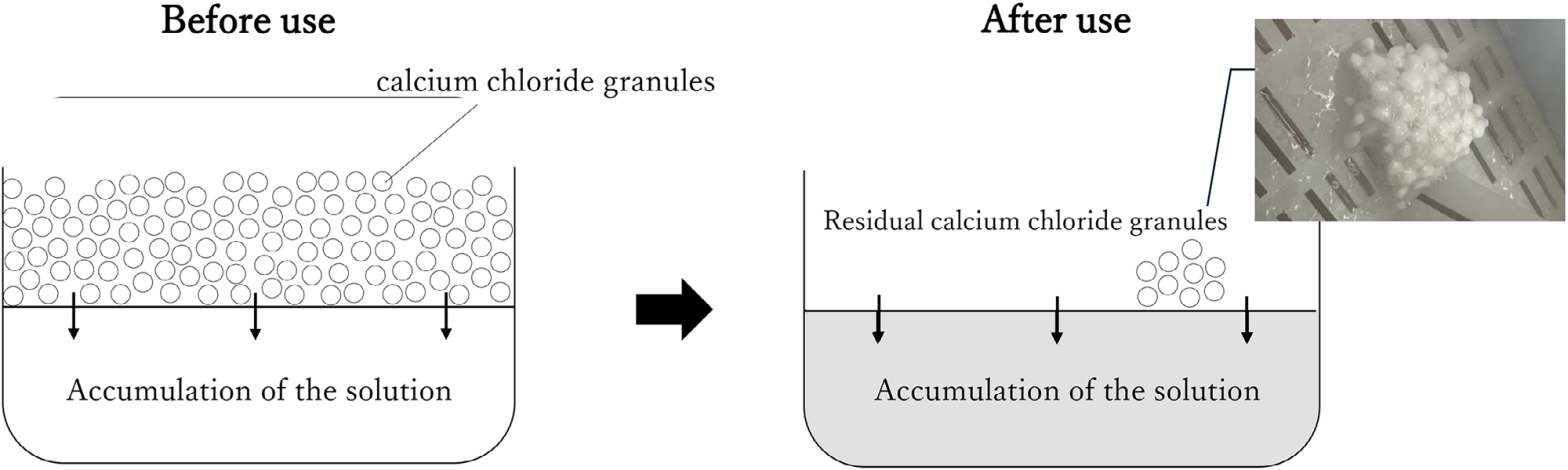
Product ingested by the patient. The upper section of the desiccant container contains calcium chloride granules, which undergo a chemical reaction upon contact with moisture, gradually absorbing water and converting into liquid. The resulting solution accumulates in the lower section of the product. The liquid had fully accumulated in the lower section; residual calcium chloride granules were still observed in solid form remaining in the upper compartment.

The severity of mucosal injury is not exclusively attributable to pH but also to factors such as electrolyte dissociation, concentration, ingested volume, and duration of mucosal contact [2]. Although severe corrosive gastritis is typically associated with strong alkalis, there are documented cases of significant injury caused by mildly alkaline substances as well. Several factors may have contributed to the severity of injury in the present case. Due to the patient's dementia, the exact amount ingested was unknown; however, symptoms began 48 h prior to admission, suggesting prolonged exposure to calcium chloride. Extended mucosal contact time is also suspected. As shown in Figure 4, the ingested desiccant consisted of an upper compartment containing calcium chloride granules and a lower compartment collecting the resulting solution formed via gradual water absorption through a chemical reaction. Once dissolved, the direct irritant effect from dehydration is diminished. However, in practical use, solid granules may remain even after activation. Although calcium chloride is usually neutralized by gastric acid, in this case, a solution containing undissolved particles was likely ingested. This may have led to delayed dissolution and prolonged mucosal exposure before complete neutralization. Furthermore, the highly acidic gastric environment likely intensified the exothermic reaction during neutralization, causing more extensive thermal injury to the stomach compared to the esophagus. Prior reports of corrosive gastritis due to calcium chloride have described cases requiring total gastrectomy or resulting in death from gastric necrosis [3, 4].

Endoscopy showed diffuse, circumferential mucosal injury in the gastric body, especially along the greater curvature, suggesting prolonged mucosal contact with partially dissolved calcium chloride granules (Figures 2 and 3). Minimal esophageal involvement likely reflects rapid passage through the esophagus. Duodenal erosion was limited, possibly due to brief exposure or partial neutralization by gastric acid. Importantly, disproportionate sparing of the esophagus with necrotic gastric or duodenal lesions may indicate caustic ingestion and help guide diagnosis. In conclusion, this case demonstrates that even mildly alkaline substances, such as calcium chloride, can cause severe corrosive gastritis if the solid components reach the stomach and remain in contact with the tissue for extended periods. This highlights the importance of considering the amount ingested, presence of solid components, and duration of exposure when evaluating patients suspected of ingesting mildly alkaline substances.

## Ethics Statement

Ethical approval was not sought for the present study because a case report is a medical activity.

## Consent

Written informed consent for publication of the clinical details was obtained from each of the patients, and a copy of each consent form is available if requested by the Editor of the journal.

## Conflicts of Interest

The authors declare no conflicts of interest.

## Data Availability

The availability of anonymized data presented in this case report can be obtained from the corresponding author (A.M.) upon motivated request.
